# Buzzing Homes: Using Citizen Science Data to Explore the Effects of Urbanization on Indoor Mosquito Communities

**DOI:** 10.3390/insects12050374

**Published:** 2021-04-21

**Authors:** Nadja Pernat, Helge Kampen, Jonathan M. Jeschke, Doreen Werner

**Affiliations:** 1 Leibniz Centre for Agricultural Landscape Research, Eberswalder Str. 84, 15374 Müncheberg, Germany; doreen.werner@zalf.de; 2 Institute of Biology, Freie Universität Berlin, Königin-Luise-Str. 1–3, 14195 Berlin, Germany; jonathan.jeschke@fu-berlin.de; 3 Berlin-Brandenburg Institute of Advanced Biodiversity Research, Königin-Luise-Str. 2–4, 14195 Berlin, Germany; 4 Friedrich-Loeffler-Institut, Federal Research Institute for Animal Health, Südufer 10, 17493 Greifswald-Insel Riems, Germany; helge.kampen@fli.de; 5 Leibniz Institute of Freshwater Ecology and Inland Fisheries, Müggelseedamm 310, 12587 Berlin, Germany

**Keywords:** biodiversity, citizen science, epidemiology, mosquitoes, urbanization

## Abstract

**Simple Summary:**

Many mosquito species can transmit pathogens and may pose a risk to human health. With increasing urbanization and alteration of natural habitats, the composition of mosquito communities is changing, with some species thriving particularly well in and adjacent to human settlements. In the present study, indoor mosquito collections submitted to the citizen science project ‘Mückenatlas’ were used to investigate the composition, abundance, and diversity of species of different urbanization levels, and to detect preferences for less or more urbanized areas. We found that species richness and diversity decreases with increasing urbanization, and some important vector species are captured most frequently in densely urbanized regions. Our results highlight the importance of long-term mosquito monitoring to learn how these vectors respond to habitat change caused by humans. Only with sufficient knowledge about the ecology of mosquitoes can we assess risks, plan counter strategies, and take action.

**Abstract:**

Urbanization has been associated with a loss of overall biodiversity and a simultaneous increase in the abundance of a few species that thrive in urban habitats, such as highly adaptable mosquito vectors. To better understand how mosquito communities differ between levels of urbanization, we analyzed mosquito samples from inside private homes submitted to the citizen science project ‘Mückenatlas’. Applying two urbanization indicators based on soil sealing and human population density, we compared species composition and diversity at, and preferences towards, different urbanization levels. Species composition between groups of lowest and highest levels of urbanization differed significantly, which was presumably caused by reduced species richness and the dominance of synanthropic mosquito species in urban areas. The genus *Anopheles* was frequently submitted from areas with a low degree of urbanization, *Aedes* with a moderate degree, and *Culex* and *Culiseta* with a high degree of urbanization. Making use of citizen science data, this first study of indoor mosquito diversity in Germany demonstrated a simplification of communities with increasing urbanization. The dominance of vector-competent species in urban areas poses a potential risk of epidemics of mosquito-borne diseases that can only be contained by a permanent monitoring of mosquitoes and by acquiring a deeper knowledge about how anthropogenic activities affect vector ecology.

## 1. Introduction

With continuing outbreaks of mosquito-borne diseases in Mediterranean countries and recent cases of West Nile fever as far north as Germany, the management of mosquito vector species has become an important political and scientific issue throughout Europe [1]. Many countries have implemented mosquito-monitoring programs based on various methodological approaches. The collected data are used to update and predict species distributions, such as tracking the spread of invasive or native mosquito species that are capable of transmitting disease agents such as dengue, chikungunya, West Nile, or Zika viruses [2,3].

Urbanization is thought to be one of the main anthropogenic drivers of changes in mosquito species composition and relative abundance through loss of natural larval habitats and the creation of new artificial ones [4,5,6]. With urbanization, an increase in population densities of those mosquito species is expected because they thrive in urban environments and in the vicinity of humans due to a selective advantage, e.g., the capability of breeding in artificial containers or the preference for human blood hosts. These include species of the genera *Aedes*, *Anopheles*, and *Culex*, some of which have invasive potential and can transmit a variety of pathogens, such as the Asian tiger mosquito *Aedes albopictus* [7]. Invasive species are highly adaptable and often prosper in urban environments, amplifying the risk of mosquito-borne disease outbreaks [8]. Consequently, it is of utmost interest and importance for risk assessment and epidemiological modelling to know which mosquito species dwell in human settlements and how mosquito communities differ based on surrounding environmental features such as the level and structure of urbanization.

Few studies about mosquito diversity in urban regions of Europe exist, with only two pertaining to metropolitan areas in Germany [9,10] and only a handful for other European countries [11,12,13,14,15,16]. By comparison, responses of mosquito communities to urbanization have been investigated more intensively in North and South America [17,18], Asia [19,20], Australia [21], and Africa [22], probably due to past or recent outbreaks of mosquito-borne diseases. The majority of these investigations focus on identifying hotspots of one or two synanthropic, highly vector-competent species such as the yellow fever mosquito *Aedes aegypti* or *Ae. albopictus* in relation to urbanization [12,23]. Studies have rarely been aimed at capturing the entire mosquito biodiversity and relating it to urbanization [17]. A key reason for this lack of studies is that access to private properties is limited. The alternative—placing traps on public land—is risky and too often results in damaged or stolen devices [24]. As a result, it is deemed necessary to include residents in the research process via a citizen science approach in order to safely collect data from around and inside homes.

Citizen science has become an increasingly common form of research over the last decade [25,26,27]. Among its many benefits for society, it facilitates data collection on a spatial and temporal scale that scientists alone are barely able to cover [28]. However, there are doubts about the explanatory power of data gathered by non-professionals, as they tend to contain observation biases such as uneven spatial coverage [29,30,31], inconsistent sampling behavior [32,33], or uncertainties in object identification by the participants [34]. On the other hand, advanced methods have been developed in recent years for each stage of the scientific process, including avoidance of bias through adapted protocols [35,36], verification of data using artificial intelligence [34,37,38], detection and statistical compensation of biases [33,39,40,41,42], and data integration [43].

Regarding urban ecology, data collected by citizens have been used in many studies, such as investigating the biodiversity of taxa like birds [44] or phorid flies [45], tracking invasive species [46], or initiating conservation action [47]. Many citizen science projects are aimed at monitoring and controlling mosquitoes as they are easily identifiable and people are personally concerned due to health implications or nuisance. In Italy, for example, a novel approach by Caputo et al. [48] used citizen surveys via an app (ZanzaMapp) to estimate mosquito abundance and nuisance. By means of the originally Spanish ‘Mosquito Alert’ smartphone app, participants could upload pictures of five important mosquito vectors and corresponding breeding habitats to inform health authorities in the Barcelona region [49]—a successful concept that has been launched in 17 other countries in 2020. Despite the relevance for public health, there is, to our knowledge, no study that explicitly focuses on the indoor biodiversity of mosquitoes. Indeed, very few studies have been conducted that target the insides of the participants’ residences, although investigating the ecology and evolution of the indoor biome is an emerging research field and is predestined for citizen science approaches [50].

The lack of knowledge about which mosquitoes actually enter human residences might be partly filled by data from the citizen science project ‘Mückenatlas’, an implemented part of the German mosquito monitoring program. To gain knowledge about the occurrence and distribution of native and invasive mosquito species, this program was initiated in 2011 and consists of several monitoring schemes such as collecting eggs by ovitrapping, larvae by dipping, and adults by placing attractant traps. This systematic approach was extended by the passive surveillance instrument ‘Mückenatlas’ in 2012, where people were asked to collect and submit mosquito samples without any protocol and training [51]. By 2020, approximately 154,000, mostly hand-caught, mosquitoes had been submitted as physical samples, with more than 66% coming from the inside of the participants’ homes, thus providing a rich data source for the current study.

This study investigates the indoor diversity of mosquitoes based on ‘Mückenatlas’ submissions from inside private homes. We take a multi-level approach to determine and specify differences of mosquito communities from varying levels of urbanization, defined by two indicator variables, *soil sealing* (surface imperviousness) and *human population density*. First, we visualize and test whether mosquito communities differ among levels of urbanization. Second, rarefied species richness and effective Shannon diversity as biodiversity indices are calculated to find explanations for the found differences. Finally, we investigate whether mosquitoes, aggregated into genera, show preferences for certain levels of urbanization. In the broader context of the uniqueness of the dataset, we simultaneously investigate whether the information contained in the data confirms our knowledge of mosquito ecology or even leads to new insights.

## 2. Materials and Methods

### 2.1. The Citizen Science Dataset

The ‘Mückenatlas’ project calls upon the German population to catch mosquitoes, kill them without damage, e.g., by freezing, and send them together with a submission form that is downloadable from the project website (www.mueckenatlas.com) to the involved institutes. Every participant is rewarded with a personal email or letter with details about the catch and, if desired, an individual marking on the collectors’ map on the project website. The institutes will morphologically and, if necessary (i.e., in ambiguous cases), genetically identify the submitted sample to species level using the identification keys of Becker et al. [52] and Schaffner et al. [53] and CO1 barcoding [54], respectively. We considered mosquito groups or complexes (e.g., *Anopheles maculipennis* complex, *Culex pipiens* complex, *Aedes annulipes* group) as single taxa to account for impossibilities or uncertainties in differentiating females between species. These complexes or groups are referred to as *species* for simplification (Supplementary Materials Table S1). All data corresponding to a mosquito submission is uploaded to the German mosquito database CULBASE.

Data were extracted from CULBASE for the years 2012 to 2019. The dataset consisted of 26,060 entries, with each entry representing one mosquito species submission from one location on a unique date, hereafter referred to as submission. One submission might contain several individuals of the same species when participants caught more than one mosquito on the same occasion; these are then summed up in an additional count variable. The exported dataset comes with an automatically generated suite of covariates, such as geo-coordinates, land-use type, and collection date. In addition, the dataset has a variable that reflects the participants’ comments on the collection location, such as garden, house, or stable etc. These were categorized manually, and all entries were then filtered according to the locations of the submissions from the interior, resulting in 16,933 observations.

### 2.2. Classification of Urbanization Level by Indicator Variables

To define the corresponding level of urbanization of every observation, we used two indicator variables: (1) percentage of sealed soil (imperviousness) and (2) population density as the number of individuals per square kilometer. Concerning sealing, we basically followed the categorization by Böcker [55] and defined a value from 0 to 50% as low, from 51 to 70% as moderate, from 71 to 90% as strong, and from 91 to 100% as very strong. A grid of the percentage of *soil sealing* related to the surface of Germany with a resolution of one square kilometer served as the data base [56], from which the corresponding value was extracted for each individual submission location and then allocated to either low, moderate, strong, or very strong sealing. In addition to *soil sealing* as a common measure for urbanization, *human population density* was considered because humans unknowingly create numerous larval habitats, e.g., in private gardens, green spaces, or cemeteries, while also providing reliable sources of blood meals, either by themselves or by their pets and their livestock. The assessment according to *human population density* was derived from the degree of urbanization classification (DEGURBA) of the EU [57], categorizing a population density of up to 300 inhabitants per square kilometer as rural, between 300 and 5000 inhabitants per square kilometer as peri-urban, and above 5000 inhabitants per square kilometre as urban. We created a human population raster with square kilometer grid cells based on data from the German census in 2011 [58], extracted the corresponding data for every submission-related collection site, and assigned categories of either rural, peri-urban, or urban (see Supplementary Materials, Figure S1, for maps on distribution of both indicator variables across Germany). For simplification, we further refer to mosquito communities by level of urbanization as groups. Data preparation and creation of spatial covariates were conducted in R version 3.6.3 [59] with packages dplyr [60], raster [61], and rgdal [62].

### 2.3. Statistical Analysis

We used non-metric multidimensional scaling (NMDS) to explore differences in mosquito community composition according to level of urbanization, a common approach to visualize multidimensional data in two-dimensional space. This ordination technique is based on ranked proximities between the subjects of interests, in this case, the abundance (submission numbers) of mosquito species and level of urbanization. Each year of data collection (2012 to 2019) was treated as a replicated sample, and the respective urbanization levels of both indicators represented the sampling units. The impact of frequently submitted species was minimized by Wisconsin double standardization and square-root transformation, and the Bray-Curtis index was used to create dissimilarity matrices based on the species submission numbers within each group. For both runs with command *metaMDS* (*vegan* package), we calculated the stress level, which is an indicator of the reliability of the result, e.g., an ordination with a stress greater than 0.3 could also have occurred arbitrarily. To test the groups for statistically significant differences in species communities, a permutational multivariate analysis of variance (PERMANOVA) was applied, followed by a pairwise comparison of groups with a permutation test based on t-statistics (homogeneity of dispersion, PERMDISP).

We chose two biological diversity metrics, rarefied species richness, and effective Shannon diversity, which are robust against varying sample sizes and abundances, and facilitate comparing differences in biodiversity between groups. Rarefaction is a standardization technique that suits the ‘Mückenatlas’ data as it accounts for the different sample sizes and allows a fair comparison between the urbanization categories. For all calculations, we used the smallest sample size for each urbanization level in each year as the number of sub-samples randomly drawn from the larger samples to estimate expected species richness (sample-based rarefaction [63] with command *rarefy* of the vegan package).

The original Shannon–Wiener index was not used, as it is difficult to interpret and not robust against differences in abundances; in our case, number of submissions and sample sizes. These disadvantages are partially resolved by using the exponential of the Shannon–Wiener index to convert it to effective Shannon diversity. It indicates the effective number of species, i.e., those that are equally common, and allows us to directly compare the results among groups [64]. Following the calculation of rarefied species richness and effective Shannon diversity, we applied ANOVA for group-wise and post-hoc Student’s t-test with the Bonferroni–Holm adjustment for pairwise comparisons.

To find out whether the citizen science data can be used to infer preferences of mosquito genera for a certain level of urbanization, a Chi-square test of homogeneity was applied. Because the number of submissions from the considered level of urbanization varied greatly, we adapted the method of Bates et al. [65] by using weighted expected counts in the Chi-square test, i.e., we calculated for each of the five genera the summed ratio of the other four genera’s observations from the different levels of urbanization to approximate the corresponding sampling effort in the expected count for the target genus. To test the single genera for significant tendencies of being submitted from certain levels of urbanization, the Chi-square residuals were computed and positive and negative tendencies visualized. These analyses were performed with R packages dplyr [60], vegan [66], ggpubr [67], and ggplot2 [68].

We opted for statistical analyses that allowed us to investigate how mosquito communities change along an urbanization gradient. For this purpose, we used species abundances for NMDS and biodiversity indices as well as abundances of genera for the Chi-square tests. A more detailed ecological examination of the occurrences of individual species, their habitat preferences, and contributions to differences in mosquito communities are beyond the scope of the current study and will be carried out in the future.

## 3. Results

**Figure 1:**
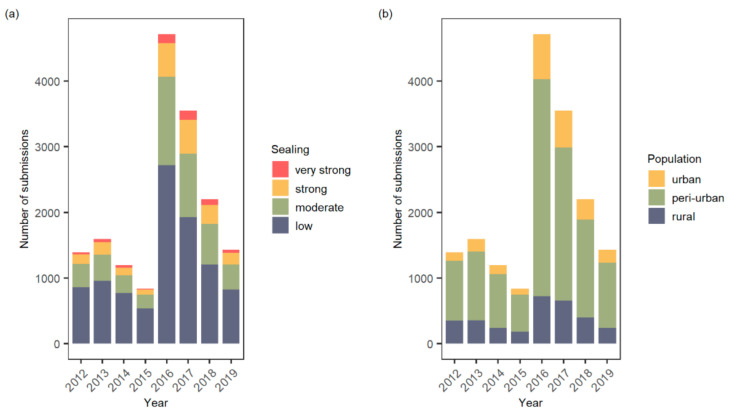
Numbers of submissions by year and level of urbanization, the latter assessed by (**a**) *soil sealing* and (**b**) *human population density*.

Distribution of submissions over years and urbanization levels varied greatly (Figure 1 and Supplementary Materials, Table S2). Most submissions were recorded in 2016 and 2017, a phenomenon based on media topicality and recorder bias that has already been investigated in previous studies [30,69]. In general, a higher number of submissions came from lower to medium levels of urbanization than from very densely populated areas. The uneven distribution across the groups according to population density (inhabitants per square km) is striking, with over two thirds of the entries coming from grid cells with 300 to 5000 inhabitants, which is not representative of the latest share of DEGURBA classes in Germany (34% rural, 42% peri-urban, 24% urban [57]).

**Figure 2:**
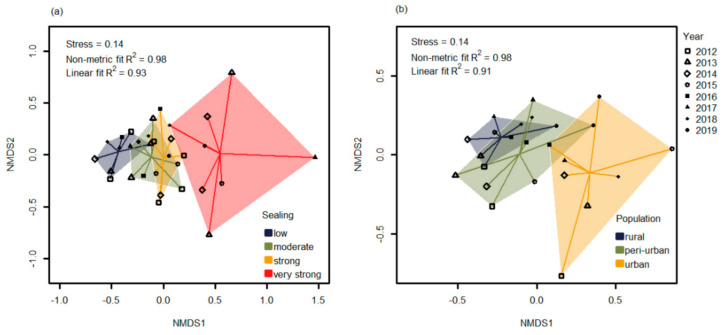
Non-metric multidimensional scaling (NMDS) showing differences in mosquito species communities of different levels of urbanization assessed by (**a**) *soil sealing* and (**b**) *human population density*, using years as replicates (symbols).

The NMDS plots show differences in yearly mosquito assemblages by groups for both indicators of soil sealing (stress value = 0.14, R^2^ = 0.98) and human population density (stress value = 0.14, R^2^ = 0.98). Stress values indicate a fairly good fit (Figure 2). Visually, the NMDS plots (Figure 2) suggest that mosquito communities of high and low urbanized areas are distinct. The PERMANOVA is significant, and the variance explained is fair for both indicator variables of soil sealing (R^2^ = 0.55, *p* < 0.001) and human population density (R^2^ = 0.44, *p* < 0.001) (Supplementary Materials, Table S3, *p*-values based on permutations).

This result might indicate that there are different species present (or present in different abundances), depending on urbanization level. Significant PERMDISP for indicator *soil sealing* suggests that, for this variable, the difference might rather be due to within-group dispersion, e.g., of greater abundance variation in the group of low sealing than in the groups of strong and very strong sealing. The case was different for the indicator *human population density*, where the PERMDISP test was not significant. To better understand these patterns, biodiversity indices were calculated.

**Figure 3:**
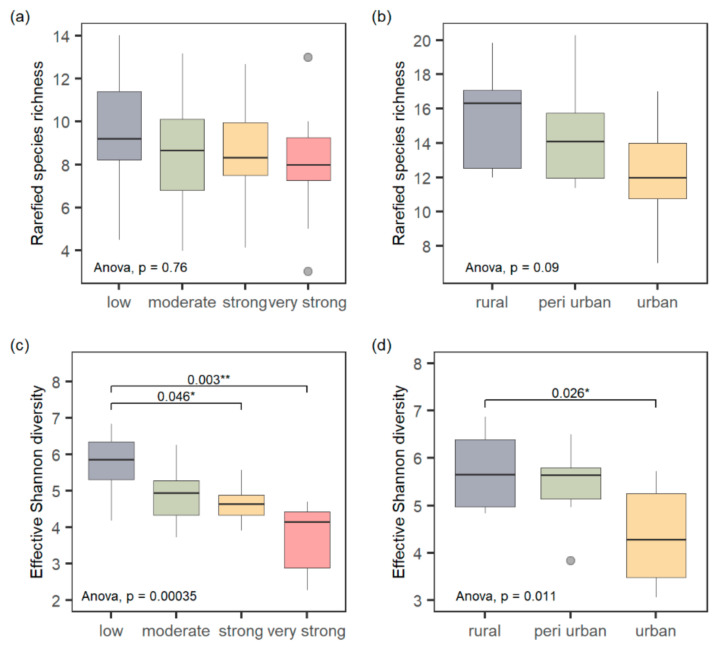
Boxplots comparing rarefied species richness, (**a**,**b**), and effective Shannon diversity (number of equally common species), (**c**,**d**), by urbanization level based on two indicators, *soil sealing* and *human population density*, using years as replicates. Thick black lines denote medians, first and third quartiles are shown by lower and upper hinges, and whiskers represent distance from hinge to the farthest value within the 1.5 interquartile range. Outliers are displayed individually. Symbols * and ** indicate statistical significance at α < 0.05 and < 0.01 based on t-tests with the Bonferroni–Holm correction (adjusted *p*-values displayed).

To explore the characteristics of the data, we plotted species richness, rarefied species richness, adjusted species richness, the Shannon–Wiener index, effective Shannon diversity, and the adjusted Shannon–Wiener index by year (Supplementary Materials, Figure S2). We then computed and visualized rarefied species richness and effective Shannon diversity per urbanization group and indicator (Figure 3). According to the ANOVA, rarefied species richness is not significantly different among groups for both indicator variables, i.e., the level of urbanization does not appear to have any influence on the number of species submitted when accounting for different sample sizes. With respect to effective Shannon diversity, we found significant differences between low and strong levels of urbanization. With a higher level of urbanization, the number of effective species decreases, i.e., there is a strong dominance of a few species (*Cx. pipiens* complex, *Culiseta annulata* and *Aedes japonicus*) in urban areas (Figure 3).

The omnibus Chi-square test revealed significant differences in the number of genera submitted per group for both indicators, *soil sealing* (χ^2^ = 80.5, *p* < 0.001) and *human population density* (χ^2^ = 159.91, *p* < 0.001). A follow-up with single comparisons (row-wise by genera to find out tendencies for level of urbanization) showed significant differences in submission numbers for most genera, except for *Culiseta*, regarding the urbanization indicator *soil sealing*, and *Coquillettidia*, regarding the indicator *human population density* (Table 1).

**Figure 4:**
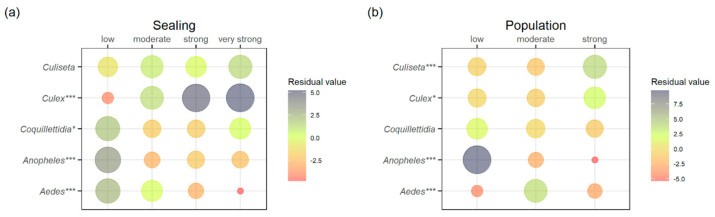
Tendencies of genera for a certain level of urbanization categorized by the indicators *soil sealing* (**a**) and *human population density* (**b**) by means of Pearson residuals. Dot size corresponds to the overall contribution to the total Chi-square value. Positive scores indicate an attraction (green to blue) and negative scores indicate a repulsion between rows and columns (yellow to red). Significant differences from row-wise comparisons are indicated (* = *p* < 0.05, *** = *p* < 0.001).

By visualizing the Pearson residuals of the Chi-square test to explore tendencies of mosquito genera for a certain urbanization level (Figure 4), a general preference of the genus *Anopheles* for rural areas, of the genera *Culex* and *Culiseta* for more densely populated environments, and of the genus *Aedes* for peri-urban spaces could be demonstrated.

## 4. Discussion

This is the first large-scale indoor mosquito biodiversity study for Germany, based on 16,933 submissions to the citizen science monitoring scheme ‘Mückenatlas’ from inside the homes of the participants between 2012 and 2019. Without the contribution of citizens, it would not have been possible to collect such data and analyze the biodiversity of mosquitoes in human housing. Therefore, citizen science seems almost a necessity for indoor biome research, at whatever scale. For example, at the lowest scale, citizens could participate simply by letting scientists into their homes so that professionals can systematically sample there (e.g., [70]). In this case, the involvement of citizens in the scientific process is extremely limited, as is the amount of data that is collected because this is highly dependent on financial and human resources. The scaling of projects can be expanded in time and space the more autonomously and flexibly citizens are involved, e.g., in physical data sampling, photorecording observations, or other parts of the scientific process [71].

However, while the flexibility of the protocol leads to a high number of participants, it also induces data bias [31]. In the case of the ‘Mückenatlas’ scheme, differences in sample size by urbanization level reflects a spatial bias, predominantly caused by population density, a phenomenon well-known from opportunistic citizen science data [29,30,33,72]. In this study, the huge differences in sample sizes within years and between the groups of urbanization level were counteracted with rather simple methods to demonstrate the general interpretability and usefulness of the opportunistic data collection for addressing ecological questions. Regardless of the biases that need to be addressed with methods according to the analysis objective, involving citizens might be the only way to get indoor biome data at all. Citizen science is also crucial for collecting a meaningful amount of information when it comes to national, continental, or even cross-continental comparative studies.

With the support of citizens providing valuable information from their homes, this study found that indoor mosquito communities differ by urbanization level. A location effect could be identified for the indicator *human population density*, whereas differences of the indicator *soil sealing* might be due to within-group dispersion, e.g., changes in relative abundances within the group over the years. By further applying biodiversity indices to shed more light on these differences, we see that the tendency that rarefied species richness decreases with increasing urbanization, as already demonstrated in smaller scale studies [11,15,73]. The higher species richness of sample aggregations stemming from rural homes appear to reflect a more heterogeneous landscape featuring habitats suitable for rarer and more specialized mosquito species. However, total species richness independent from level of urbanization varies greatly over time (Supplementary Materials, Figure S2), suggesting that fluctuating factors other than *soil sealing* and *human population density* shape the recordable diversity of species. Climatic conditions greatly influence the development and composition of mosquito communities [74,75,76] and can lead to higher densities, nuisance, and media topicality, thus increasing the probability of a submission by a ‘Mückenatlas’ participant [30]. In addition, the opportunistic data collection is not only biased by human population and climatic variability but also by taxonomic preferences [32]. In the case of citizen science programs where both native and invasive taxa are of interest, people tend to look out for the intruders [77,78]. Therefore, species richness estimates from citizen science data need to be carefully interpreted and, if possible, combined or cross-checked with professional data [79,80].

Comparison of effective Shannon diversity also indicates that diversity decreases with urbanization, thereby supporting the results of rarefied species richness estimates and partly explaining the significant difference of the groups. Although a meta-study by Fenoglio et al. [81] found hematophagues to be the only group of arthropods that generally seems to positively respond to urban environments, mosquito communities are less diverse in populated and sealed areas [11,73,82]. While the disturbance of natural habitats through deforestation or drainage of wetlands negatively affects the life cycle of rather specialized species, adaptive generalists are promoted by urbanization. Indeed, some of the most competent vectors of the *Aedes*, *Culex*, and *Anopheles* genera show tendencies to exploit edges of disturbance such as forest-arable land transitions, abandoned stables, or construction sites at urban expansion borders [21,83]. The tendency of submitted *Aedes* specimens to be collected predominantly in peri-urban areas could therefore also be due to high submission numbers of *Ae. japonicus*, a species that also prefers these transition zones, to the ‘Mückenatlas’ scheme [84].

Of all mosquito genera, *Culex* is the most frequent in urban and strongly sealed areas, mainly due to the high numbers of *Cx. pipiens* complex submissions. Members of the *Cx. pipiens* complex are ecologically and physiologically flexible and are known to thrive in urban areas [85,86]. They reproduce as easily as other urban-adapted mosquito species in widely available artificial containers [87]. As such, artificial containers offer microhabitats that enable mosquito species to survive despite dry seasons or droughts [88]. Even the emergence of the human-biting preference of *Ae. aegypti* or a shift of breeding site selection by the minor malaria vector, *Anopheles plumbeus*, towards man-made habitats can now be attributed to adaptation to urban regions with reliable water sources [88,89,90].

## 5. Conclusions

Our results demonstrate that citizen science is an appropriate method in the process of analyzing the indoor biome and, moreover, that the ‘Mückenatlas’ opportunistic data collection not only confirms existing knowledge but also enables completely new insights into urban mosquito ecology. Although the analysis is greatly simplified by combining all submissions and creating artificial groups of mosquito communities, regardless of the geographical or climatic conditions of the original location, the explanatory power of the data is strong—certainly due to the large observation number. Citizen science is therefore not only recommended for inclusion in formal mosquito monitoring programs to enlarge the data basis for better risk assessments and modelling, it could also unleash a truly invaluable resource that can significantly advance the global indoor biome data—the people at home [91].

The results of this study are also relevant for public health in Germany. The high submission numbers of *Cx. pipiens* complex from within people’s homes and from high levels of urbanization (i.e., densely populated areas) highlight the risk of human exposure to mosquito-borne disease in the country. The simplification of mosquito communities in urban areas worldwide, as confirmed by our study, is caused by less differentiation of breeding sites through homogenization of urban habitats, which is in turn linked to higher infection rates [11,73]. Initial natural diversity would not recover, even over a century after being urbanized [17], so the natural mechanism of reducing species-related nuisance through intraspecific competition will not be restored. In the face of accelerated urbanization and global warming, precautions can only be taken with further intensive surveillance and knowledge acquisition. Therefore, continuous mosquito monitoring on large scales, even cross-national, with conventional methods and citizen science are just as essential as targeted small-scale field studies to achieve a better understanding of vector ecology.

## Figures and Tables

**Table 1:** Chi-square test of homogeneity for the number of observations per urbanization indicator—*soil sealing* and *human population density*—of five mosquito genera. Expected counts are weighted by the proportion of samples of the four other genera (ns = not significant).

Genus	Observed Counts				Weighted Expected Counts				χ^2^	*p*-Value
**Sealing**	**Low**	**Moderate**	**Strong**	**Very Strong**	**Low**	**Moderate**	**Strong**	**Very Strong**		
*Aedes*	2386	1064	427	86	2268	1063	489	143	36.61	<0.001
*Anopheles*	464	149	65	12	397	187	83	23	28.23	<0.001
*Coquillettidia*	238	79	31	12	208	97	43	12	11.21	<0.011
*Culex*	4424	2150	1023	300	4706	2092	877	222	70.40	<0.001
*Culiseta*	2297	1102	480	144	2341	1073	482	128	3.70	ns
**Population**	**Rural**	**Peri-Urban**	**Urban**		**Rural**	**Peri-Urban**	**Urban**			
*Aedes*	645	2830	488		738	2683	543		4.18	<0.001
*Anopheles*	233	413	44		128	467	94		131.24	<0.001
*Coquillettidia*	80	239	41		67	244	49		41.81	ns
*Culex*	1462	5311	1124		1470	5346	1081		71.62	<0.028
*Culiseta*	732	2670	621		749	2723	551		8.51	<0.001

## Data Availability

For the time being, mosquito data used in the submitted manuscript cannot be provided publicly as it would violate the personal privacy of the citizen scientists.

## Supplementary Materials

The following is available online at https://www.mdpi.com/article/10.3390/insects12050374/s1, Table S1: Species list with corresponding numbers of submissions to the ‘Mückenatlas’. Table S2: Total counts of submissions by year and level of urbanization; Table S3: PERMANOVA results based on Bray–Curtis dissimilarities using square-rooted abundance data for indoor mosquito communities grouped by (a) *soil sealing* and (b) *human population density*; Figure S1: Distribution of indicator categories across Germany; Figure S2: Differences in the biodiversity indices with or without consideration of the sampling effort across all years, regardless of urbanization indicator. Figures S1 and S2 were produced in R [59].
