# Supplementary material for: Buzzing Homes: Using Citizen Science Data to Explore the Effects of Urbanization on Indoor Mosquito Communities
Source: Insects. 2021 Apr 21;12(5):374. doi: 10.3390/insects12050374 (PMC8143366; doi:10.3390/insects12050374)
Supplement: Supplementary file 1 [file insects-12-00374-s001.zip › insects-1144033-supplementary.pdf]

Table S1: Species list with corresponding numbers of submissions to the 'Mückenatlas'.

Species	No. of submissions
Ae. aegypti	1
Ae. albopictus	54
Ae. annulipes group	337
Ae. caspius	73
Ae. cataphylla	63
Ae. cinereus/geminus/rossicus group	40
Ae. communis	13
Ae. detritus	13
Ae. dorsalis	1
Ae. flavescens	6
Ae. geniculatus	980
Ae. intrudens	2
Ae. japonicus	858
Ae. koreicus	1
Ae. leucomelas	22
Ae. pullatus	4
Ae. punctor	15
Ae. refiki	1
Ae. rusticus	73
Ae. sticticus	433
Ae. vexans	973
An. claviger complex	45
An. maculipennis complex	396
An. petragnani	1
An. plumbeus	248
Cq. richiardii	360
Cs. alaskaensis	1
Cs. annulata/subochrea	3933
Cs. glaphyroptera	7
Cs. longiareolata	6
Cs. morsitans/fumipennis	76
Cx. hortensis	22
Cx. modestus	16
Cx. pipiens complex	7837
Cx. territans	22

Table S2. Total counts of submissions by year and level of urbanisation.

	**Urbanisation by sealing**				**Urbanisation by population**			
	low	moderate	strong	very strong	rural	peri-urban	urban	**total**
2012	863	353	143	33	350	916	126	1392
2013	959	397	193	49	358	1043	197	1598
2014	773	268	118	37	240	819	137	1196
2015	535	213	80	12	180	567	93	840
2016	2720	1348	507	143	723	3307	688	4718
2017	1928	967	514	143	659	2332	561	3552
2018	1207	618	290	87	400	1488	314	2202
2019	824	380	181	50	242	991	202	1435
**total**	9809	4544	2026	554	3152	11463	2318	**16933**

Table S3. PERMANOVA results based on Bray-Curtis dissimilarities using square-rooted abundance data for indoor mosquito communities grouped by a) soil sealing and b) human population density.

PERMANOVA

Urbanisation indicator		Df	SumofSq	Pseudo-F	R^2	p-value
Sealing						
	Groups	3	1.9766	11.29	0.5474	0.001***
	Residuals	28	1.6340		0.4526	
	Total	31	3.6106		1.0000	
Population						
	Groups	2	0.7582	8.248	0.4399	0.001***
	Residuals	21	0.9651		0.5601	
	Total	23	1.7233		1.0000	

PERMDISP

Urbanisation indicator		Df	SumofSq	Mean Sq	Pseudo-F	p-value
Sealing						
	Groups	3	0.0411	0.0136	2.9615	0.041*†
	Residuals	28	0.1294	0.0046		
Population						
	Groups	2	0.0117	0.0059	1.9526	0.159
	Residuals	21	0.0631	0.0030		

[1] Df: Degrees of freedom; SumofSq: sum of squares; Pseudo-F: F-value based on 999 permutations; p-valu: based on 999 permutations (lowest P-value possible: 0.001), †significant differences in permutated p-values for the soil sealing category pairs 'low'-'very strong' and 'moderate'-'very strong'.

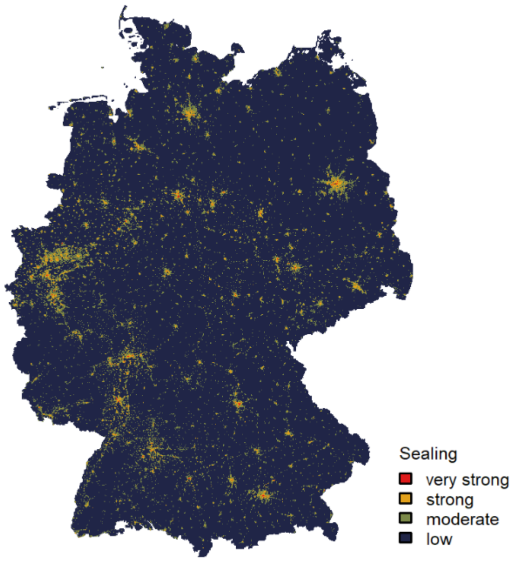

Figure S1. Distribution of indicator categories across Germany. Raster grid with cell size of one square kilometre for soil sealing (left) and human population density (right).

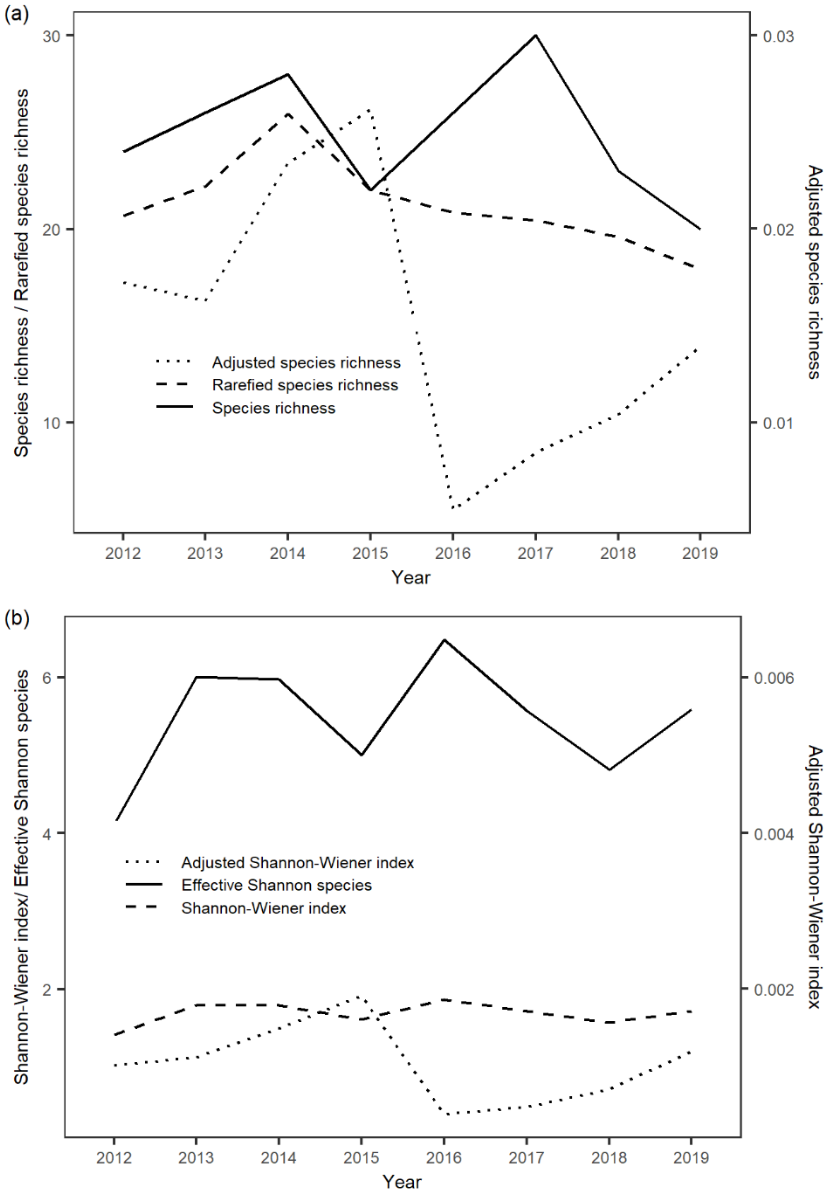

Figure S2. Differences in the biodiversity indices with or without consideration of the sampling effort across all years, regardless of urbanisation indicator. (a) Species richness, rarefied species richness (based on the smallest sample size of 840 submissions in 2015) and adjusted species richness (species richness divided by the respective number of submissions per year) (b) Shannon-Wiener-Index, effective Shannon diversity and adjusted Shannon-Wiener index (Shannon-Wiener index divided by the respective number of submissions per year). Figure calculation and design based on [44].
